# A global scale COVID-19 variants time-series analysis across 48 countries

**DOI:** 10.3389/fpubh.2023.1085020

**Published:** 2023-04-27

**Authors:** Rachel Yui Ki Chu, Kam Chiu Szeto, Irene Oi Ling Wong, Pui Hong Chung

**Affiliations:** ^1^School of Public Health, LKS Faculty of Medicine, The University of Hong Kong, Hong Kong, Hong Kong SAR, China; ^2^Department of Finance, Business School, The Chinese University of Hong Kong, Hong Kong, Hong Kong SAR, China

**Keywords:** COVID-19, variant of concern (VOC), strategy, global, time-series, mutation

## Abstract

**Background:**

The coronavirus disease (COVID-19) pandemic is slowing down, and countries are discussing whether preventive measures have remained effective or not. This study aimed to investigate a particular property of the trend of COVID-19 that existed and if its variants of concern were cointegrated, determining its possible transformation into an endemic.

**Methods:**

Biweekly expected new cases by variants of COVID-19 for 48 countries from 02 May 2020 to 29 August 2022 were acquired from the GISAID database. While the case series was tested for homoscedasticity with the Breusch–Pagan test, seasonal decomposition was used to obtain a trend component of the biweekly global new case series. The percentage change of trend was then tested for zero-mean symmetry with the one-sample Wilcoxon signed rank test and zero-mean stationarity with the augmented Dickey–Fuller test to confirm a random COVID trend globally. Vector error correction models with the same seasonal adjustment were regressed to obtain a variant-cointegrated series for each country. They were tested by the augmented Dickey–Fuller test for stationarity to confirm a constant long-term stochastic intervariant interaction within the country.

**Results:**

The trend series of seasonality-adjusted global COVID-19 new cases was found to be heteroscedastic (*p* = 0.002), while its rate of change was indeterministic (*p* = 0.052) and stationary (*p* = 0.024). Seasonal cointegration relationships between expected new case series by variants were found in 37 out of 48 countries (*p* < 0.05), reflecting a constant long-term stochastic trend in new case numbers contributed from different variants of concern within most countries.

**Conclusion:**

Our results indicated that the new case long-term trends were random on a global scale and stable within most countries; therefore, the virus was unlikely to be eliminated but containable. Policymakers are currently in the process of adapting to the transformation of the pandemic into an endemic.

## Introduction

The World Health Organization (WHO) declared the SARS-CoV-2, commonly known for causing COVID-19, a global pandemic crisis on 11 March 2020 (1). According to the WHO, as of 16 September 2022, in total, there were 608,328,548 confirmed cases and 6,501,469 claimed deaths (1). It had been over 2.5 years since the declaration, and this epidemiological crisis has remained a controversial issue worldwide. Numerous challenges

came along with the pandemic. Scientists and researchers across the world were working around the clock to invent vaccines and strategies for curing COVID-19. Despite researchers across the world continuing to investigate the strategies to end the pandemic, including vaccination promotion, vaccine effectiveness toward variants of concern (VOCs), and quarantine effectiveness (2, 3), the pandemic was still ongoing.

The long fight against COVID-19 had led to an economic downturn, as many countries had imposed lockdowns, which had hugely limited global human mobility. In addition to the economic aspects, the healthcare system had been given extra resources and burdens (4). The WHO had provided operational guidance for maintaining essential health services while enhancing medical surveillance to contain the spread of COVID-19 (5). This has resulted in a large number of patients being affected and delayed their medical appointment schedules (6). Furthermore, countries are facing a dilemma in balancing the COVID-19 response and essential healthcare services. Various viewpoints were raised by different countries, with some of them insisting that preventive measures should be upheld while others preferred the world to return to normal without strict preventive measures against the virus. In this study, we aimed to investigate whether there is an uncontrollable, random trend of global new cases and to identify whether the COVID virus behaved like the influenza virus to be long-living and seasonally fluctuating with different VOCs (7) or as a one-off outbreak like severe acute respiratory syndrome (SARS) (8).

## Method

### Data extraction and processing

Daily reported new case numbers, and the ratio of major concerns of variants per each country from 02 May 2020 to 29 August 2022 were acquired from the GISAID database (https://ourworldindata.org/grapher/covid-variants-bar) (9). There were 10 time series representing the ratio of major concerns of variants per each country, Alpha, Beta, Gamma, Delta, Omicron (BA.1), Omicron (BA.2), Omicron (BA.4), Omicron (BA.5), Omicron (BA.2.12.1), and Omicron (BA.2.75), and 1 extra for other variants. The data were resampled on a biweekly basis due to data sparsity of variant ratio data. There were at most 61 data points per country. The missing data points were filled by the previous data point, which extended for at most 1 month, assuming that the monthly variations were not significant. Only countries with processed data for over two-thirds of the period, i.e., at least 41 data points, were analyzed to avoid misinterpretation of the results. A total of 48 countries met the inclusive criteria after filtering: Argentina, Australia, Austria, Bangladesh, Belgium, Brazil, Canada, Chile, Croatia, Czechia, Denmark, Estonia, Finland, France, Germany, Greece, Hong Kong, India, Indonesia, Ireland, Israel, Italy, Japan, Kenya, Latvia, Lithuania, Luxembourg, Malaysia, Mexico, Netherlands, Norway, Peru, Philippines, Poland, Portugal, Romania, Russia, Singapore, Slovakia, Slovenia, South Africa, South Korea, Spain, Sweden, Switzerland, Turkey, the United Kingdom, and the United States. Ratios of the submitted sequence of COVID variants of each country were then multiplied with the biweekly reported new case numbers of each country, yielding the expected number of biweekly new cases by a variant type for each country.

### Long-term trends of worldwide biweekly new cases

By adding up the biweekly new cases of the included countries, a worldwide biweekly new case series was obtained. It was decomposed for the seasonal component analysis to analyze its trend, seasonality, and noise to remove the seasonality property of COVID (10). An additive model would be used if the series was homoscedastic (serial independence in regression residual) by time progression, which would be verified by the Breusch–Pagan test (11). Otherwise, a multiplicative model would be used (12). The cycle period was set at seven time steps, assuming a regular quarterly spatiotemporal fluctuation (13).


$$\begin{array}{l} \{\begin{array}{c} \begin{array}{c} y\left(t\right)=T\left(t\right)+S\left(t\right)+\epsilon \\ y\left(t\right)=T\left(t\right)\times S\left(t\right)\times \epsilon \end{array}\begin{array}{c} additive\;model\\ \;multiplicative\;model \end{array} \end{array} \end{array}$$

where *y*(*t*) represented the worldwide biweekly new case series to be decomposed, *T*(*t*) was the trend component representing the long-term progression of the series, *S*(*t*) was the seasonality component representing the regular seasonal variation of the series, and ϵ was the residual noise.

In this study, the convolution method (linear kernel) was used to filter the trend and seasonality components (14). The trend component was extracted by the following moving average formula:


$$\begin{array}{l} \begin{array}{c} T\left(t\right)=\frac{1}{2\times 7\;+\;1}\overset{7}{\underset{i=-7}{\sum }}x_{t+i}\;for\;t>7 \end{array} \end{array}$$

Then, by removing the trend component from the observed data, the seasonal component was obtained by the mean of every 7th data point from the detrended data starting from the 1st, 2nd, 3rd, 4th, 5th, 6th, and 7th data points:


$$\begin{array}{l} S\left(t\right)=\frac{1}{n\left(A\right)}\underset{i\in A}{\sum }\left[x_{i}-T\left(i\right)\right] \end{array}$$

where


$$\begin{array}{l} \begin{array}{c} A=\left\{i\;:i\;mod\;7=t\;mod\;7\;for\;1\leq i\in \mathbb{N}\leq n\left(X\right)\right\}\\ n\left(A\right)=\#of\;items\;in\;A \end{array} \end{array}$$

The remaining unexplained component by trend and seasonality of the observed data was considered residual noise.

The regressed trend component *T*(*t*) was extracted as the smoothened series representing the trend of the worldwide biweekly new cases for studying the long-term trend. The percentage change per time step of this trend was tested with the one-sample Wilcoxon signed-rank test for zero-mean symmetrical distribution and augmented the Dickey–Fuller test for stationarity, with no lag-level difference allowed and a non-deterministic trend. We could identify the characteristics of the change of trend component to observe if a random or drifted long-term trend of the number of worldwide biweekly new cases existed.

### Cointegration between expected new case numbers by variants within countries

Variants of concern (VOCs) were studied by individual countries. To verify if there exist any cointegration relationships between the time series of the estimated number of reported cases of different variants of each shortlisted country, i.e., if the case-by-variant series would have a long-term constant stochastic trend, a Vector Error Correction Model (VECM) with one seasonal lag level (7 data points) was regressed for the case of each country, under the hypothesis that there exist (1) at least one cointegration rank with case numbers by variants and (2) a constant long-term trend of case numbers such that the seasonal difference was a zero-mean normal variable.


$$\begin{array}{l} {\Delta y}_{t}=\phi_{0}+\Pi_{y_{t-1}}+\Phi {\Delta y}_{t-1}+\epsilon_{t}\\ {\Delta y}_{t}=y_{t}-y_{t-7} \end{array}$$

where *y*_*t*_ was the case-by-variant vector at time *t*, ϕ_0_ was the regressed intercept term as the case-by-variant vector at time 0, Π_*y*_*t*−1__ was the error correction term, Φ was the coefficient vector of the auto-correlated case-by-variant term for time *t*−1, and ϵ_*t*_ was white noise at time *t*. The error correction term Π_*y*_*t*−1__ can be decomposed into αβ^*T*^, where β could be extracted as the cointegrating vector for stationary testing (15).

The normalized dot product of the estimated cases by variants of each country and the corresponding cointegrating vector was obtained as the “cointegrated series” of the country. This series was tested by the augmented Dickey–Fuller test for stationarity, with no lag-level difference allowed and under a non-deterministic trend assumption. A stationary “cointegrated series” confirmed a constant long-term stochastic trend in the number of the combination of cases by variants of that particular country.

All data manipulation, visualization, modeling, and testing were carried out by Python 3.9.7 under Jupyter notebook environment, with the aid of the Pandas, NumPy, Matplotlib, SciPy, and Statsmodel libraries. The alpha values of all statistical tests were set as 0.05.

## Results

### Long-term trends of worldwide biweekly new cases

**Figure 1:**
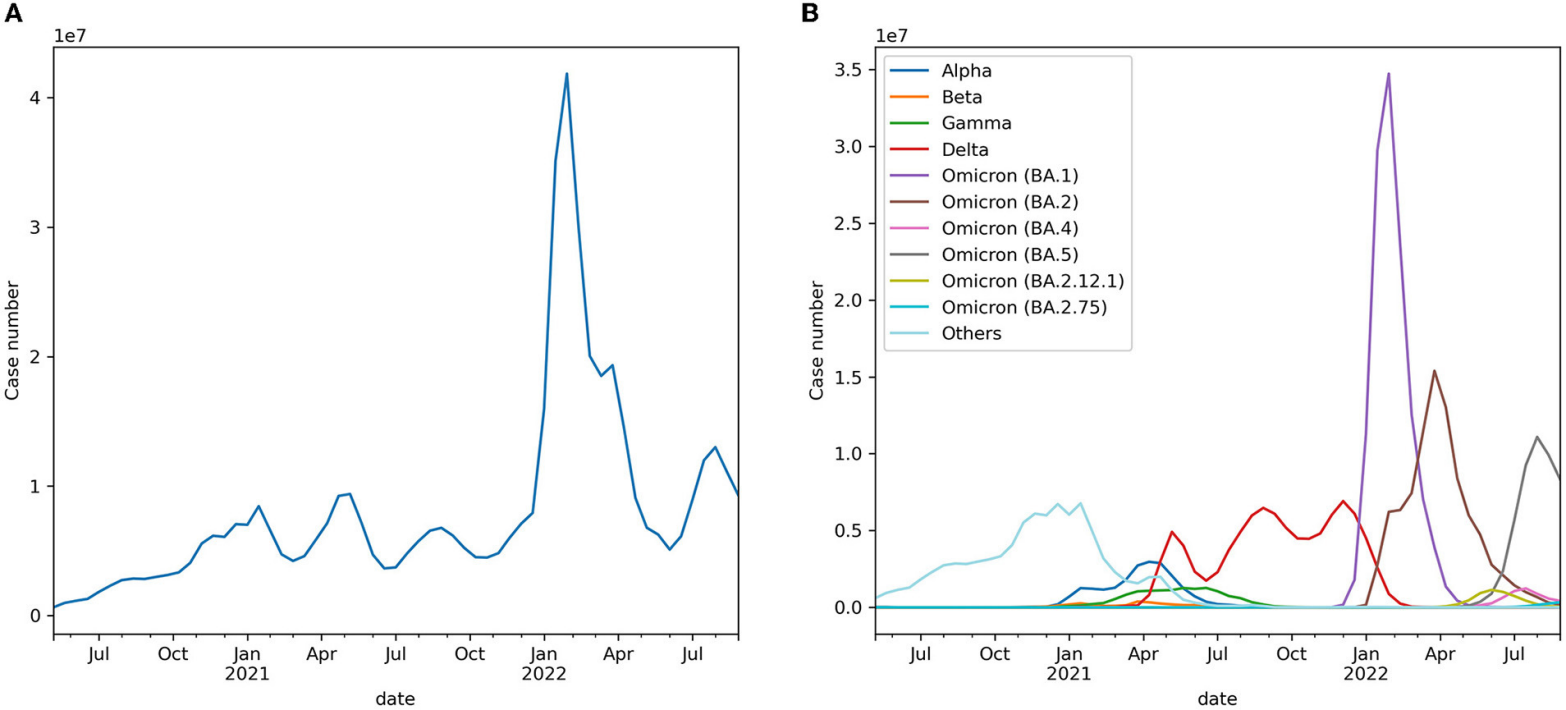
**(A)** Expected worldwide biweekly new cases. **(B)** Expected worldwide biweekly new cases by variants.

The trend of biweekly new cases over the globe and their expected compositions by variants are displayed in Figure 1. It was observed that the biweekly new case series had no deterministic or directional trend, except for the sudden surge in Omicron (BA.1 & BA.2) in early 2022. The Breusch–Pagan test results showed that the series was heteroscedastic (*p* = 0.002).

**Figure 2:**
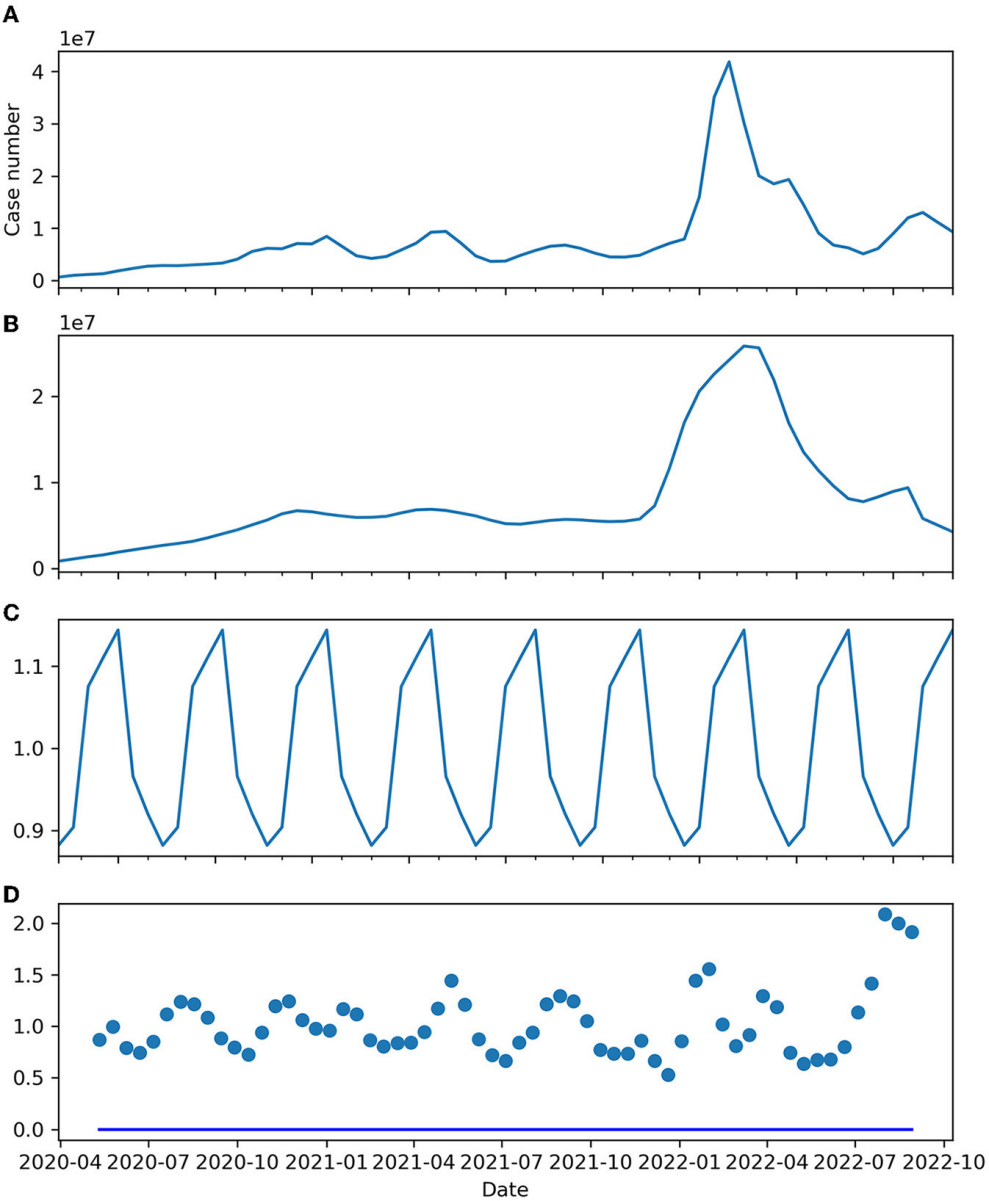
**(A)** Expected worldwide biweekly new cases. **(B)** Trend component. **(C)** Seasonal component. **(D)** Residual.

**Figure 3:**
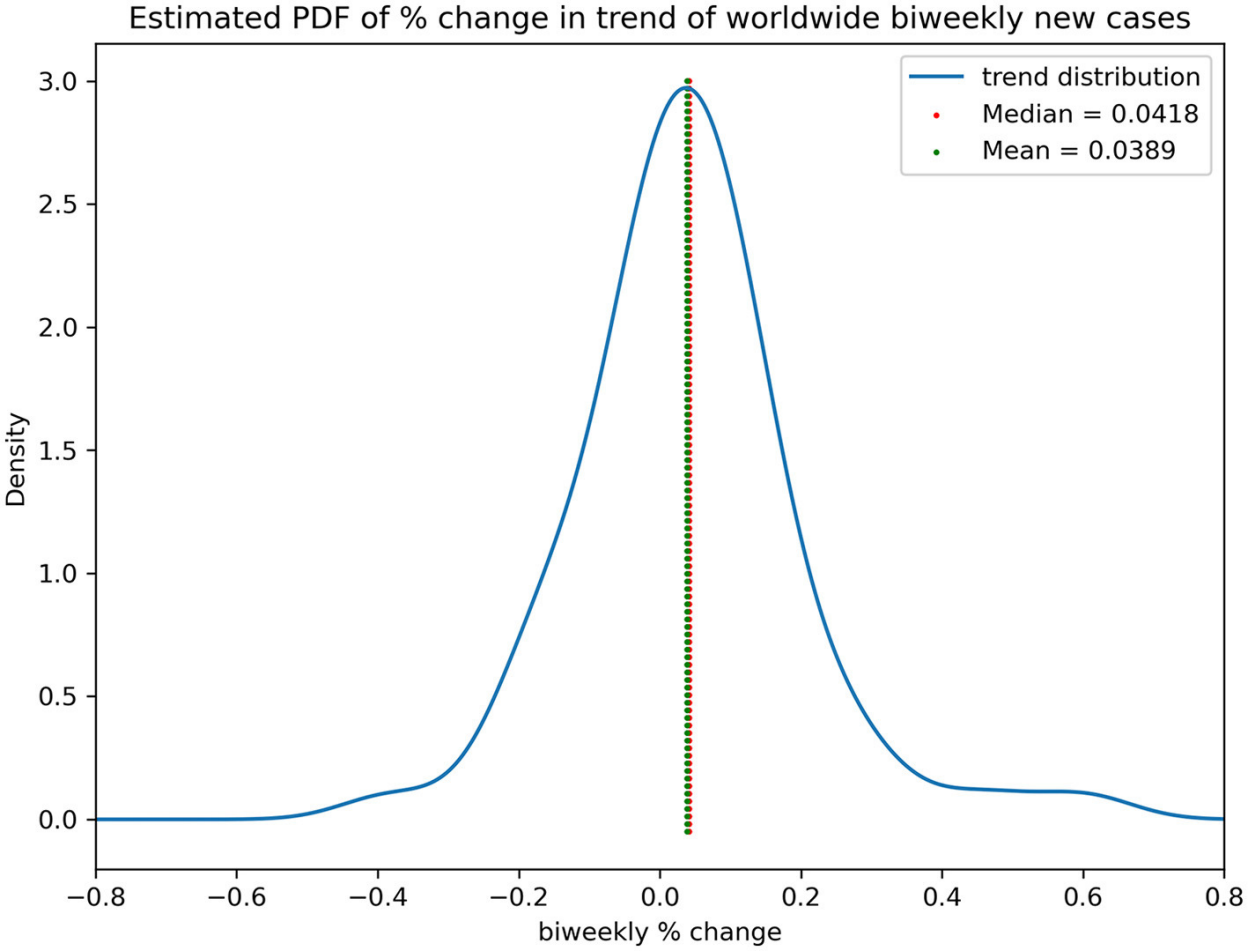
Estimated PDF of % change in trend of worldwide biweekly new cases.

As observed in a near-quarterly cyclic fluctuation, the series was decomposed into a trend, seasonal and residual components, as shown in Figure 2, *via* a multiplicative model. The mean and variance of the percentage change in the smoothened trend component were 0.039 (+/– 0.155) but were symmetrically distributed in terms of zero-mean (*p* = 0.052) and non-deterministically stationary (*p* = 0.024). Figure 3 shows the estimated probability density function of the percentage change in the smoothened trend component. A heavy tail on the positive side might have skewed the distribution, explaining why the distribution was only weakly symmetric, given the *p*-value was very close to the threshold of rejecting the null hypothesis.

### Cointegration between expected case numbers by variants within countries

**Figure 4:**
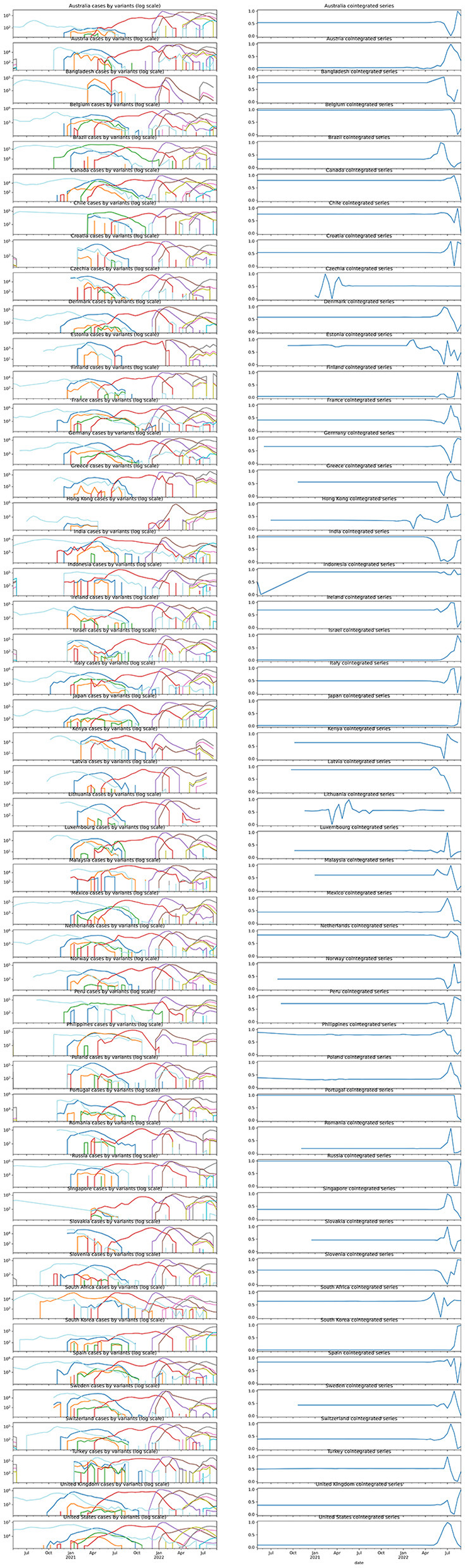
Case-by-variant by country and their corresponding normalized cointegration series.

Table 1 and Figure 4 show that 37 out of 48 countries had their case-by-variants cointegrated. The cointegrated series of most countries were very stationary until some stirrings were observed in early- and mid-2022, which was the period when worldwide Omicron (BA.1 & BA.2) cases surged, but most of them returned to original levels afterward.

**Table 1:** Stationarity test on cointegration series by country.

**Country**	***p*** **-value**	**Cointegrated**
Argentina	0.000000	✓
Australia	0.000029	✓
Austria	0.161500	
Bangladesh	0.008982	✓
Belgium	0.422995	
Brazil	0.001473	✓
Canada	0.984466	
Chile	0.000000	✓
Croatia	0.000000	✓
Czechia	0.000000	✓
Denmark	0.008688	✓
Estonia	0.000012	✓
Finland	0.000001	✓
France	0.000002	✓
Germany	0.000013	✓
Greece	0.000004	✓
Hong Kong	0.000019	✓
India	0.110762	
Indonesia	0.000001	✓
Ireland	0.000015	✓
Israel	0.683367	
Italy	0.000000	✓
Japan	1.000000	
Kenya	0.000000	✓
Latvia	1.000000	
Lithuania	0.000000	✓
Luxembourg	0.000000	✓
Malaysia	0.002662	✓
Mexico	0.009539	✓
Netherlands	0.735694	
Norway	0.000000	✓
Peru	0.000001	✓
Philippines	0.369270	
Poland	0.002056	✓
Portugal	0.935441	
Romania	0.000000	✓
Russia	0.000068	✓
Singapore	0.011175	✓
Slovakia	0.000000	✓
Slovenia	0.000828	✓
South Africa	0.000000	✓
South Korea	0.974641	
Spain	0.000000	✓
Sweden	0.000002	✓
Switzerland	0.000095	✓
Turkey	0.000037	✓
United Kingdom	0.001633	✓
United States	0.044868	✓

## Discussion

### Interpretation of research findings

Our results discovered that the percentage change in the underlying trend of the biweekly new case series was a zero-mean symmetrical distribution. The series was heteroscedastic, but meant differently for variance by time. However, the direction of the trend was indeterministic. This increasing randomness over time was very likely coming from the Omicron spike during early 2022. This development suggested that there existed random long-term biweekly COVID new case numbers after seasonal adjustment, which was difficult to model by any distribution. Given the percentage change in the symmetrical zero-mean distribution with non-deterministic variance, one might simulate the trend series by a Heston model (16). It was the collective result of all governments' interventions, people's actions, and environmental factors. As a result, it was likely that the trend in COVID new cases was uncontrollable, random, and unlikely to be diminished by human interference.

However, there were 77.1% of the involved worldwide nations, regardless of their geographical locations, side of the hemisphere, major ethnicity, or population structure and density, exhibited a relatively constant and stable seasonality-adjusted cointegration relationship between different VOCs. A recent study suggested that a variant would dominate a period and subside but then will be replaced by another strand in its ratio of all COVID new cases (17). Our study provides an additional quantitative proof of not only the ratio of the VOCs but also of their newly infected numbers which behaved in that way, resulting in seasonal fluctuation but consistent COVID infection numbers that never ended. The variant-cointegrated countries had a wide range of stringent measures in COVID response policies (18). This could suggest that the strength of COVID control might be able to control the virus spread but not the existence of the virus, as no measure could be taken to prevent the rise of a new variant. The different properties between global and regional scales suggested that the inconsistent policies between countries made the infection uncontrollable, whereas local consistent strategies could contain the spread, regardless of their extent.

It was noticeable that only the Omicron outbreak during early-to-mid 2022 had cause a significant shock in the cointegration series in most countries. That was likely due to the unusually high infectivity and transmitting ability of the VOC (19), causing the infection numbers to ramp up and down sharply. The unsmooth transition of dominating VOC thus disrupted the balance temporarily.

### Policies implication

COVID-19 has added an extra burden to the medical system in every country regardless of preventive measures, medical expenses, and research development. Millions of individual lives have been claimed from all walks of life. We are all desperate for a cure to end this pandemic and achieve a healthy community. Owing to the enormous infection numbers and exposure to antigens due to vaccine administration (20), and from our results, the transmission of COVID-19 possibly stayed in a relatively loosely controllable range. Based on our results, over 70% of the country was cointegrated while the VOC continued to surge, and the infection control implemented within a region is sufficient for containment of the disease spread. These measures might be covariates that affect the seasonal property of the disease spread and the infection rate in some regions (10). However, these local interventions remained random in the long-term global biweekly new cases. Thus, extreme preventive measures were unlikely to control the infection number to its aim of total elimination. These results aligned with the results of previous studies (20). Elimination in the community might not be worthwhile given the large amount of medical and social resources allocated. Policymakers should be aware of this issue to balance public health concerns and economical activities.

As stated by the WHO in a media briefing on 14 September 2022, COVID-19 will continue but the wild pandemic situation is coming to an end (21). Our results serve as a quantitative proof of the statement. Our results indicated that the virus appeared to be continuing regardless of the scale and strictness of the implemented infection control policies, but the effectiveness of intracountry containment shall be appreciated. Regional infection control measures and personal hygiene should be sustained to contain the spread. However, further or upgraded anti-virus implementation including lockdowns may not be effective in containing the virus. At the same time, countries might shift their focus from eliminating the virus to avoiding seasonal outbreaks threatening the local healthcare systems. It might also add indications to the direction of preventive measures, especially those measures that are related to vaccine research. Instead of focusing on the current variants of concern, it might be useful to predict and select a few possible virus strands that might be susceptible to a possible outbreak for vaccination, just like the influenza virus.

There are several strengths to this study. This research provided quantitative proof and perspectives on the current trend, seasonal, and cointegration properties of the COVID-19 new case series. Unveiling the underlying structure, it served as a guide to an early adaption of a possible transformation from the COVID pandemic into a regular respiratory endemic. We also pinpointed the need for shifting the policy focus from tackling the current COVID-19 situation to preventing future unknown new variant outbreaks.

The data themselves could also be concerning. Since there were only at most 61 data points per country, the regressed models were sensitive to sharp changes such as the Omicron surge in early-to mid-2022. From Figure 2D, we were able to observe a heteroscedastic, periodic fluctuation in residual noise, which could be due to insufficiency in the decomposition model to capture the full feature of the underlying seasonal signals. Thus, the found trend and seasonal properties are uncertain due to unmodeled factors. Improvement in modeling the decomposition, as well as continued observation, is needed for consolidating the evidence and conclusion. In addition, the data were logged under voluntary input in the database and might not be able to fully reflect the actual ratios of variants. While no data from any African country were available after processing, they aggregated the lack of input data issue. This would affect the representativeness of the data to cover the world's situation.

## Conclusion

In this study, a random long-term trend of biweekly global new COVID cases was identified with a seasonal property. There existed cointegration relationships of newly reported cases of different variants of concerns for most countries, regardless of their demographics and responses toward the virus. The results suggested that consistent strategies could contain the spread. In addition, extreme eliminatory measures may not be effective, and a high possibility of the COVID pandemic was transforming into an endemic.

## Data availability statement

The original contributions presented in the study are included in the article/supplementary material, further inquiries can be directed to the corresponding author.

## Author contributions

RC and KS contributed to the conceptualization, data curation, methodology, writing of the original draft, contributed to the writing and editing, and investigation. PC contributed to editing and supervision. IW contributed to editing and data curation. All authors contributed to the article and approved the submitted version.
